# Signal-Conditioning Block of a 1 × 200 CMOS Detector Array for a Terahertz Real-Time Imaging System

**DOI:** 10.3390/s16030319

**Published:** 2016-03-02

**Authors:** Jong-Ryul Yang, Woo-Jae Lee, Seong-Tae Han

**Affiliations:** 1 Converged Medical Device Research Center, Korea Electrotechnology Research Institute (KERI), Ansan-si, Gyeonggi-do 15588, Korea; net10226@keri.re.kr; 2 Electric Propulsion Research Center, KERI, Changwon-si, Gyeongsangnam-do 51543, Korea; lwj0501@keri.re.kr

**Keywords:** THz system, CMOS detector array, real-time imaging, plasmon detector, electrical modulation, responsivity calibration, DC offset cancellation, signal conditioning block

## Abstract

A signal conditioning block of a 1 × 200 Complementary Metal-Oxide-Semiconductor (CMOS) detector array is proposed to be employed with a real-time 0.2 THz imaging system for inspecting large areas. The plasmonic CMOS detector array whose pixel size including an integrated antenna is comparable to the wavelength of the THz wave for the imaging system, inevitably carries wide pixel-to-pixel variation. To make the variant outputs from the array uniform, the proposed signal conditioning block calibrates the responsivity of each pixel by controlling the gate bias of each detector and the voltage gain of the lock-in amplifiers in the block. The gate bias of each detector is modulated to 1 MHz to improve the signal-to-noise ratio of the imaging system via the electrical modulation by the conditioning block. In addition, direct current (DC) offsets of the detectors in the array are cancelled by initializing the output voltage level from the block. Real-time imaging using the proposed signal conditioning block is demonstrated by obtaining images at the rate of 19.2 frame-per-sec of an object moving on the conveyor belt with a scan width of 20 cm and a scan speed of 25 cm/s.

## 1. Introduction

Terahertz (THz) waves, which are located in the frequency band between optical and electronic techniques, have very low energy levels and thus do not pose any ionization hazard [1]. These days, THz imaging is becoming a promising technology for many applications in the field of security screening and non-destructive testing of materials because it can safely obtain non-invasive images with high resolution from distant objects [2]. Especially in the application of foreign body detection in food, the imaging system is quite useful for testing all the samples in production facilities and simultaneously distinguishing food from soft foreign materials, such as bucks, carbides, and plastics, which is not possible with the X-ray imaging [3].

Real-time capabilities and a large projection area are essential for building the imaging system for practical applications [3,4]. Compared to other methodologies for terahertz detection, Si-based plasmonic CMOS detector offers more in terms of lower cost, higher sensitivity, broader spectral range, and faster temporal response [5,6]. However, application of a CMOS detector array for a terahertz imaging system has not been put to use as much as expected for three following reasons: First, detection performances are limited by low responsivity, signal-to-noise ratio (SNR), and effect of DC offsets in the detector [5]. Second, there are the inhomogeneities of the detector and the signal conditioning block, which is used for amplifying the output signals and filtering out noises [6]. Third, more often than not, a large-scale array requires rapid signal processing for real-time imaging [7].

This work presents the 0.2 THz real-time imaging system employing a 1 × 200 CMOS detector array, the signal of which is processed by the conditioning block. A pixel size of CMOS array detector should be comparable to its wavelength so that the width of the array detector for real-time imaging at sub-THz is usually bigger than single 6-inch wafer. The devices on the same wafer show the maximum 20% characteristic variation in MOSFET and the maximum 30% characteristic variation in resistors [8]. Therefore, the fabrication variation of pixels is inevitable. Consequently, it is not easy to realize the CMOS plasmon detector array with uniform dynamic range over wide area.

In this paper, we propose the signal conditioning block to compensate for the difference between the pixels with wide detector array. The conditioning block takes full advantage of gate biases of detectors and voltage gains of lock-in amplifiers to normalize the dynamic range of the pixels. The SNR of the detector array is improved by the lock-in amplifiers using the electrical modulation at the gate bias. The periodic calibration process at the output of the signal conditioning blocks enhances the uniformity of the output of the detector pixel and cancels the effect of the DC offset. The process in the signal conditioning block includes the control of the gate bias of each detector and the adjustment of the voltage gain of each amplifier in the signal conditioning block. Moreover, the block utilizes multi-channel synchronous analog-to-digital converters (ADCs) for high-speed and simultaneous signal acquisition of 200 outputs of the detector array in a projection area in real-time. The sub-THz imaging system using the proposed signal conditioning block is demonstrated with objects moving on the conveyor belt being captured in real-time at scan rate of 19.2 frame-per-sec (FPS). The architecture of the proposed THz imaging system employing the proposed conditioning block is shown in Section 2. The setup of the real-time imaging measurement by the THz system is demonstrated in Section 3.

## 2. Signal Conditioning Block for THz Imaging System

**Figure 1:**
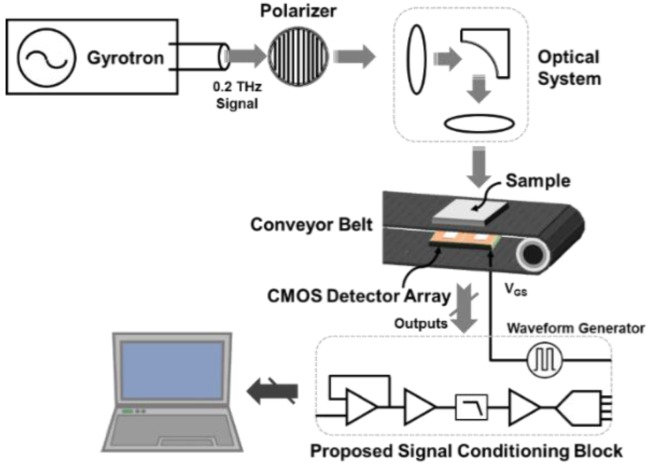
Block diagram of the real-time terahertz (THz) imaging system employing the proposed signal conditioning block of a 1 × 200 CMOS detector array.

Figure 1 shows the architecture of the real-time imaging system operating at 0.2 THz. The intensity of input signals generated by the gyrotron is determined by the polarizer because the THz beam is linearly polarized parallel to the linear polarization of the integrated patch antenna of the detector [9]. The overall intensity of the THz input coupled to the antenna of the detector can be controlled in a biquadratic cosine by changing the angle of the polarizer [6]. The THz beam distributed in Gaussian profile is converted to a uniform line beam by the optical system [10,11,12]. The optical system consists of a high-density polyethylene cylindrical lens and a metal cylindrical mirror [11]. A 1 × 200 CMOS detector array receives THz signals through samples on the conveyor belt with a moving speed of 25 cm/s. The length of a scan line is 20 cm, which is calculated by multiplying 200 pixels by a pitch of 1 mm. The detector pixel in the array, which is integrated with a linearly polarized patch antenna and fabricated by 65-nm CMOS technology, employs an asymmetric structure in the source and drain to improve the responsivity [5].

**Figure 2:**
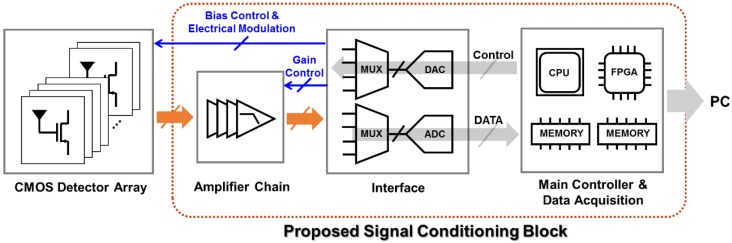
Block diagram of the overall system architecture of the proposed signal conditioning block.

**Figure 3:**
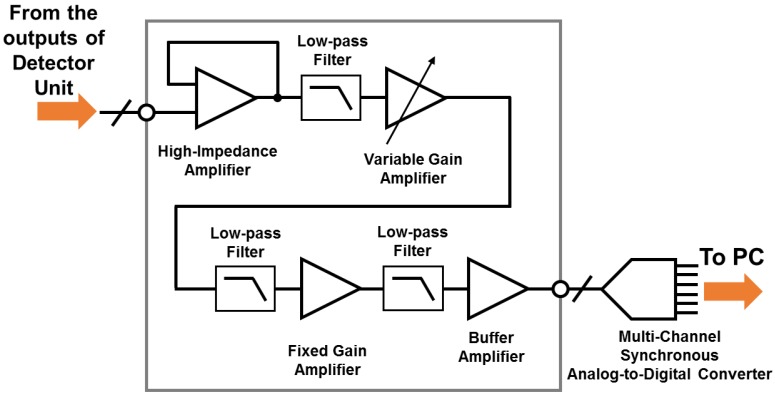
Block diagram of the the amplifier chain and interface from a detector unit to an analog-to-digital converters (ADC) in the signal conditioning block.

The signal conditioning block consists of the amplifier chain, interfaces between analog and digital signals, and the main controller and data acquisition block. The overall system architecture of the proposed signal conditioning block is shown in Figure 2. DC outputs of the detector pixels are amplified up to the voltage gain of 50 dB and filtered with a series of low-pass filters inside the amplifier chain. The signals are then collected at a main controller and data acquisition board having passed through the interface board where 13-channel synchronous 12-bit ADCs and thirteen 32 × 1 multiplexers (MUXs) reside. Figure 3 shows the block diagram of the amplifier chain, a part of the signal conditioning block for amplifying the outputs and filtering out noises in the outputs.

### 2.1. Electrical Modulation Using the Gate Bias of the Detector Pixel

A plasmon detector receives THz signals from the gate coupled to the integrated antenna and produces a DC voltage to the drain depending on the incident power of the THz signals [4,6,13]. The SNR of the detector array plays an important role in obtaining clear and accurate THz images [14]. The SNR of a single detector pixel can be improved at the chopping frequency by using a mechanical chopper and a lock-in amplifier because its performance is limited by the 1/f noise and DC offset near DC or low frequency range [7]. However, the chopper cannot be used in real-time imaging system with the large detector array because the chopper stability affects the quality of the imaging, it is difficult for the chopper covering the array with a large area to increase the chopping frequency, and the vibration of the chopper blade makes noises and decreases the uniformity of the output.

**Figure 4:**
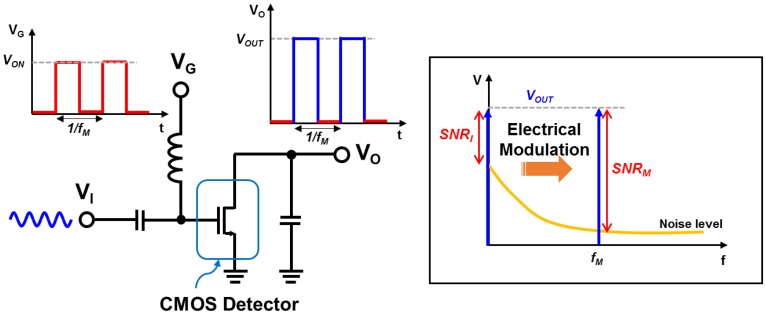
Schematic and conceptual diagram of electrical modulation of the detector pixel in the THz imaging system.

The electrical modulation by the proposed signal conditioning block is used to improve the SNR of the THz imaging system. As shown in Figure 4, the electrical modulation controlling the bias voltage of the detector can achieve the same effect as the mechanical chopper. The CMOS sub-THz detector in the array shows different output level depending on the gate bias, and the output of the detector is modulated in accordance with the gate bias [5]. The frequency of the modulation signal from the waveform generator can be controlled from 100 Hz to 1 MHz.

### 2.2. Calibration of the Detectors in the Array

**Figure 5:**
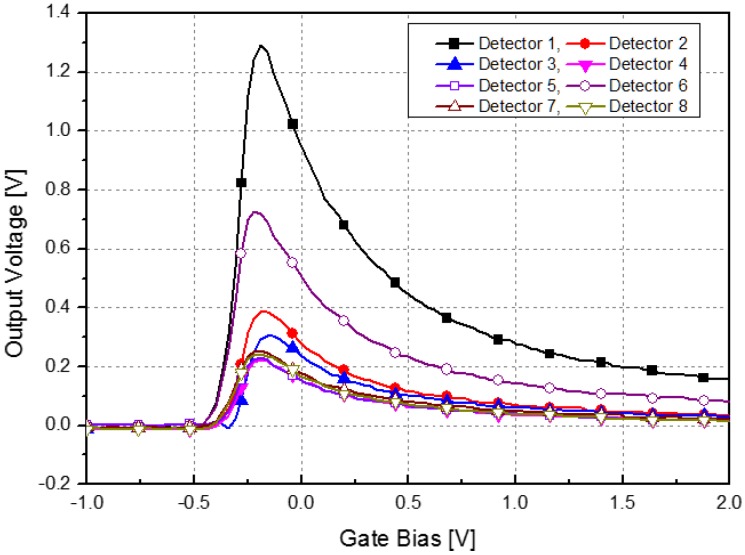
Eight examples of measured response voltages at the output among detector pixels in the array.

It is important to obtain the uniform image quality of the large-scale real-time THz imaging system in order to minimize the performance variation across the detector pixels in the array [6]. Especially, measured responsivities should be normally distributed over the pixels along the array [15]. As for an extreme case, Figure 5 shows the differences in the measured voltage responsivities of CMOS plasmon detectors consisting of the detector array due to the fabrication variation [16]. The gain variation of the amplifiers in the signal conditioning block also affects the uniformity of the imaging system [17]. The calibration process by the proposed signal conditioning block is to approximately adjust the output level of each detector pixel to the maximum input voltage of the ADC. The proposed conditioning block controls the gate bias of the detector pixels and the voltage gain of the amplifiers when the THz wave is evenly incident on the detector array for the calibration. The detectors in the array are classified with six operating conditions as shown in Table 1 after the calibration process. This process can simultaneously calibrate both the responsivity of the detector and the performance variation of the amplifier chain in the signal conditioning block. The ratio between the standard deviation and the average of the measured response voltages is 0.56 before the calibration, and it is modified to 0.02 after calibration [16]. The output signals through the amplifier chain are synchronously converted to the digital domain using thirteen ADCs, which have the maximum input voltage of 5 V and a spurious free dynamic range of −85 dB.

**Figure 6:**
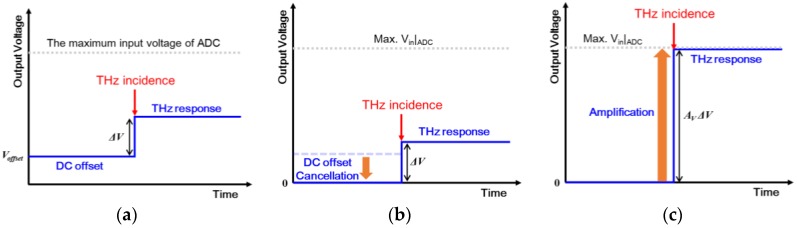
DC offset cancellation and output signal amplification in digital domain: (**a**) An output voltage of the detector pixel before DC offset cancellation; (**b**) An output voltage of the same pixel after cancellation; (**c**) An output voltage with gain control of the amplifier in the signal conditioning block.

DC offsets should be minimized because they may introduce dead pixels in the THz images constructed by the DC output voltages of the detector array. There are various sources of DC offsets in the system and an actual environment. The DC offset in each pixel can be obtained using a metal sample, which blocks THz signals generated from the gyrotron. DC offsets are stored as a reference and subtracted from the output signals in the digital domain as shown in Figure 6. Uniform THz images can be achieved from the calibration process after the cancellation.

## 3. Experiment Demonstration

**Figure 7:**
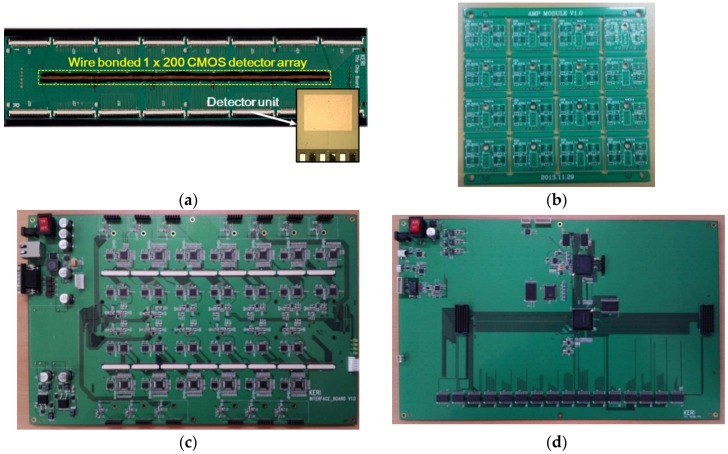
Photographs of (**a**) a 1 × 200 CMOS detector array board (size: 50 mm × 220 mm); (**b**) printed circuit boards (PCBs) for amplifier chain in the signal conditioning block (PCB unit size: 30 mm × 21 mm); (**c**) an interface board including ADCs, DACs, and multiplexers (MUXs) (size: 350 mm × 220 mm); and (**d**) a main controller and data acquisition board (size: 350 mm × 220 mm).

Module boards of the detector array and the signal conditioning block are shown in Figure 7. The detection module in Figure 7, which is fixed on the conveyor system, consists of a Teflon cover, module housing, and four printed circuit boards (PCBs) as follows: the 1 × 200 CMOS detector array board (Figure 7a), the amplifier chain board (Figure 7b), the interface board including ADCs, DACs, and MUXs (Figure 7c), and the main controller and data acquisition board for data acquisition, operation control, and communication with the PC (Figure 7d). Electrical modulation, calibration, and DC offset cancellation are operated by control signals from the main controller and data acquisition board. Parasitic characteristics are present in the CMOS detector array board because the detector unit is connected to the PCB with gold bond wires, but they are effectively reduced by using a metallic shield [6].

**Figure 8:**
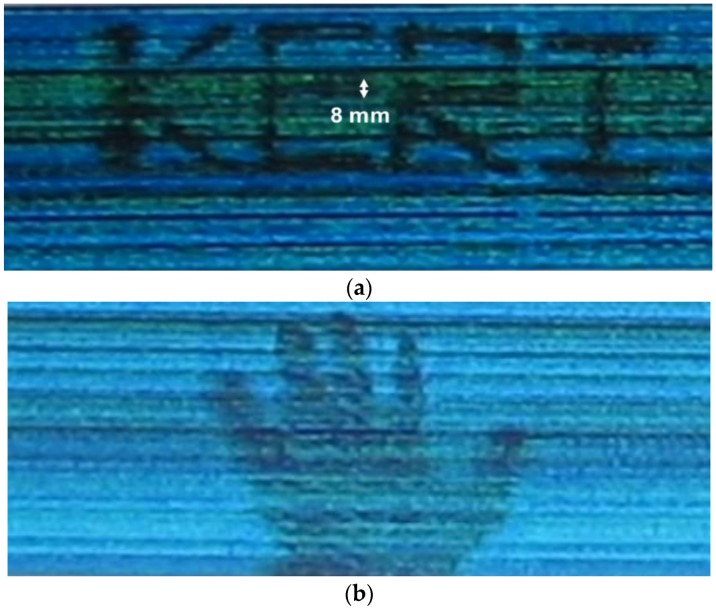
Captured image of the real-time THz video: (**a**) The institution logo, KERI (Korea Electrotechnology Research Institute); (**b**) A hand. Samples move horizontally with the scan speed of approximately 10 cm/s over the detector array. The image resolution less than 8 mm is verified from the measurement results because the measured images can discriminate the minimum line width of 8 mm marked in (a).

The performance of the proposed signal conditioning block for the CMOS detector array is demonstrated by the real-time THz images. First, the system using the proposed signal conditioning block obtains the THz images without the conveyor belt to measure the THz detection of the large-scale detector array. Samples move horizontally with the scan speed of approximately 10 cm/s over the detector array. The measured THz images of the logo of our institute and a hand are shown in Figure 8. The images are captured from the real-time THz videos. The institution logo, KERI (Korea Electrotechnology Research Institute), is made of copper tape with the thickness of 0.06 mm and attached on the polystyrene board with the dielectric constant of 1.03 and the thickness of 10 mm. The image resolution less than 8 mm is verified from the measurement results because the measured images can discriminate the minimum line width of 8 mm shown in Figure 8a.

**Figure 9:**
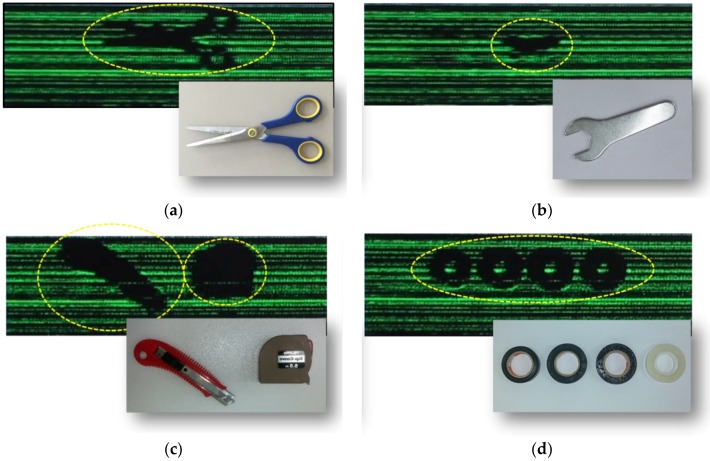
Captured images of THz real-time videos for various samples and photographs of the samples: (**a**) Scissors; (**b**) A wrench; (**c**) A pocket knife and a tapeline; (**d**) Adhesive tapes. Insets: Photographs of the samples in each image.

The next demonstration is to obtain the THz real-time images for objects with various materials on the conveyor belt with the scan speed of 25 cm/s. Test samples are transported by the conveyor belt with a moving part made of Teflon material. Figure 9 shows real-time images for various samples on the conveyor belt. THz waves are reflected from the surface of the metal, and the metal objects can be detected using the imaging system in real time as shown in Figure 9b. Plastics and adhesive tapes with a PVC can be also detected using the imaging system in real time because the THz signals are quite attenuated in highly dense plastics and tapes.

**Figure 10:**
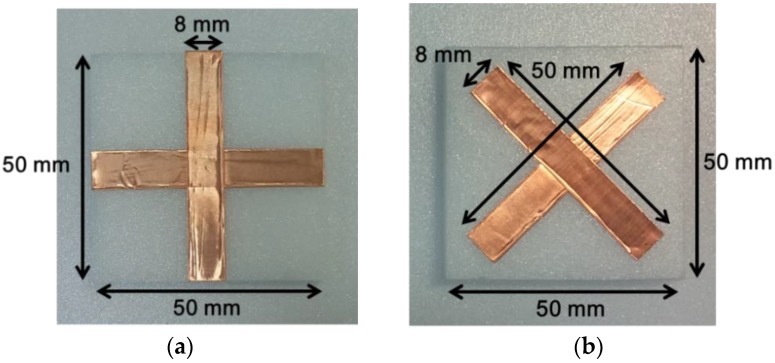
Photographs of two samples for the demonstration of the real-time THz imaging system. (**a**) the pattern of “+” ; and (**b**) the pattern of “−“.

Two types of samples are prepared for the demonstration of the real-time THz imaging system. The patterns of “+” and “−“ with the line width of 8 mm are shaped by using copper tape with the thickness of 0.06 mm and placed between two polystyrene blocks with the dielectric constant of 1.03 and the thickness of 10 mm. Figure 10 shows the detail dimension of the samples. Nine samples are arranged with the space of 20 mm on the conveyor belt.

**Figure 11:**
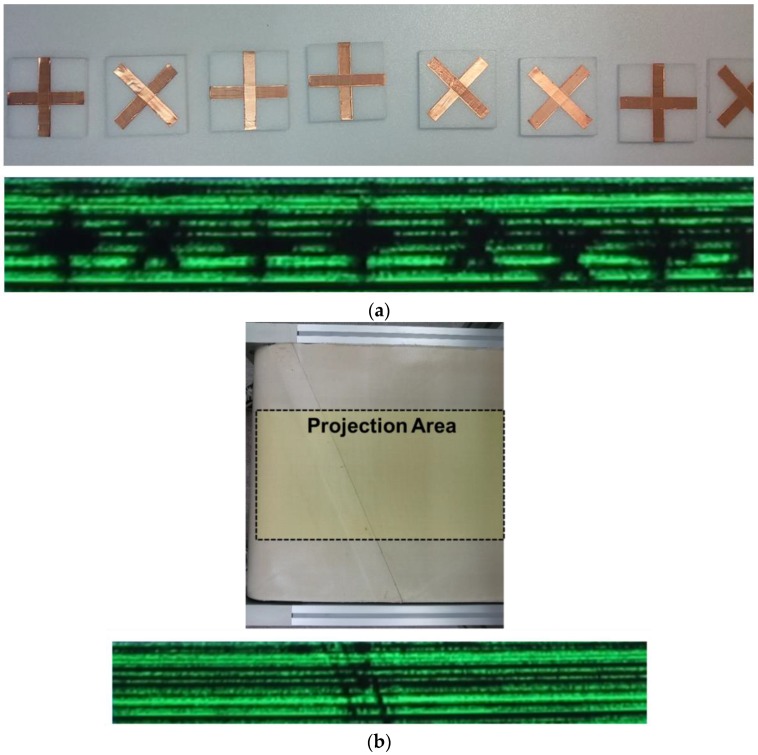
Photographs of samples and captured images of real-time THz videos of moving samples (**a**) two types of patterns of “+” and “−“ and (**b**) adhesive tapes with an acetate to bond the conveyor belt.

The patterns of “+” and “−“ are successively remarked by the imaging system using the proposed conditioning block in Figure 11a. The images are the other examples showing the image resolution of 8 mm or less. There are no image shapes for the polystyrene blocks in Figure 11a. Most THz signals are transmitted without any reflections on the surface of the blocks because the difference of dielectric constant between the air and the polystyrene blocks is negligible. The shape of adhesive tape with an acetate, which are used to bond the conveyor belt, is imaged by the system as shown in Figure 11b. In contrast to the copper tape, the THz waves can be transmitted through the adhesive tape with the thickness of 0.1 mm and the dielectric constant of approximately 5. However, the waves are attenuated by the adhesive tape because of boundary condition between the air and the tape. Figure 11b shows that the imaging system can detect the difference of signal power between the THz waves passing through the adhesive tape and otherwise.

The frame-per-sec (FPS) of the imaging system can be calculated from signal acquisition time and the number of acquisition for overall detectors in the array and expressed as

(1) $$\text{FPS}=\frac{1\text{ Frame}}{T_{S}\cdot N}$$

where *T_S_* is the period of the signal sampling and *N* is the number of acquisition needed to achieve one frame [18,19]. *N* also indicates the maximum number of detector pixels supported in the system. *T_S_* is set to 125 μs in the proposed system. The calculated *N* is 416 because the thirteen synchronous ADCs operate 32 times repeatedly by controlling the MUXs. Using Equation (1), the FPS of the proposed imaging system brings out 19.2. *T_S_* can be reduced to 100 μs, and 24 FPS can be achieved by the system. The performances of the THz imaging system using the proposed signal conditioning block are compared with the previous results in Table 2. The main difference from the previous results listed in Table 2 is the imaging method. The linear array detector in the THz imaging system obtains images over an objects continuously moving on the conveyor belt. Therefore, it carries wide fabrication-variation of the detectors. To make the variant outputs from the detector in the array uniform, the proposed signal conditioning block calibrates the responsivity of each pixel by controlling the gate bias of each detector and the voltage gain of the lock-in amplifiers in the block.

Several black lines, attributed to damaged detector pixels, are shown in the captured images of real-time THz videos. 200 CMOS detector units are separately attached on the PCB and three wires in each detector are connected to the detector units with the PCB. For that reason, the detector module consisting of a large number of the detector units is apt to cause breakage and contamination in the fabrication process. The number of the black lines is increased in Figure 9 and Figure 11 compared with Figure 8. It can be understood that the THz waves are attenuated by the conveyor belt and the outputs of the detector pixels are saturated more easily with the calibration process for the compensation of the signal attenuation.

## 4. Conclusions

We demonstrated the performance of the proposed signal conditioning block making the variant outputs from the 1 × 200 CMOS plasmon detectors in wide array uniform, by taking terahertz images in real time. The electrical modulation improves the SNR of CMOS detectors, and the calibration process with DC offset cancellation also improves the uniformity of THz images to minimize the performance discrepancy caused by the detector array and electronic circuits in the proposed signal conditioning block. The imaging system with the proposed conditioning block for 1 × 200 CMOS detector array achieved 19.2 FPS real-time imaging of the samples on the conveyor belt with the scan speed of 25 cm/s.

## Figures and Tables

**Table 1:** Operating conditions of detectors in the signal conditioning block after the calibration process.

Conditions	Gate Bias (V)	Overall Voltage Gain (dB)	Dynamic Range ^1^
1	1.0	25	4.88
2	0.2	29	4.72
3	0.1	28	4.73
4	−0.1	28	4.88
5	0.5	26	4.7
6	−0.1	27	4.84

^1^ The difference between the maximum and minimum voltages.

**Table 2:** The comparison of the THz imaging system.

Performances	[4]	[15]	[20]	[21]	This Work
Detector Process	CMOS	CMOS	CMOS	Sb-HBD	CMOS
Operating Frequency	650 GHz	280 GHz	650 GHz	700 GHz	200 GHz
Scanning Method	No scanning	2D step scan	Parallel scanning	No scanning	Line scanning
Optics	Integrated silicon lens	Lens-less	Discrete Picarin & PTFE lenses	Integrated silicon lens	Discrete polyethylene lens
# of detectors in the array	32 × 32	4 × 4	3 × 5	80 × 64	1 × 200
One projection area	4.2 cm × 4.2 cm	1 mm × 1 mm	0.2 mm × 0.15 mm ^1^	8.5 cm × 6.8 cm ^2^	1 cm × 20 cm
Imaging method	Single shot	Pixel multiplexing	Pixel multiplexing	Single shot	Continuous imaging
Scan speed		0.63 cm/s			25 cm/s
FPS	25			5	19.2
Dynamic Range		55 dB		15 dB	14 dB

^1^ The projection area is the physical area of a pixel which is used for measuring the detector performances; ^2^ The projection area was calculated with the THz images shown in the paper.

## Abbreviations

The following abbreviations are used in this manuscript:

CMOS: Complementary Metal-Oxide-Semiconductor
DC: Direct Current
SNR: Signal-to-Noise Ratio
ADC: Analog-to-Digital Converter
DAC: Digital-to-Analog Converter
MUX: Multiplexer
PCB: Printed Circuit Board
FPS: Frame-Per-Second
Sb-HBD: Antimonide Based Heterostructure Backward Diode
